# Efficacy of NAMPT inhibition in T-cell acute lymphoblastic leukemia

**DOI:** 10.1371/journal.pone.0324443

**Published:** 2025-06-17

**Authors:** Chelsea Vrana, Matthew Zhang, Max Rochette, Michelle Alozie, Hailey Oviedo, Alan Gonzalez, Jaden Sherman, Barry Zorman, Pavel Sumazin, Karen R. Rabin, Jacob J. Junco

**Affiliations:** Texas Children’s Cancer Center, Department of Pediatrics, Baylor College of Medicine, Houston, Texas, United States of America; China Medical University (Taiwan), TAIWAN

## Abstract

Novel agents targeting upregulated signaling pathways are needed to improve outcomes in T-cell acute lymphoblastic leukemia (T-ALL), since conventional cytotoxic chemotherapy regimens have reached the limits of tolerability. We identified upregulated, targetable signaling pathways common to both human T-ALL samples and a *Kras*^*LSL-G12D/+*^.*Mb1*^*Cre/+*^ murine model of T-ALL. We found the NAMPT inhibitor FK866 had the greatest cytotoxicity of a panel of small molecule inhibitors tested in human and mouse T-ALL cell lines, and in patient derived xenograft (PDX)-expanded T-ALL patient samples. We subsequently tested FK866 *in vivo* in PDX mouse models of T-ALL, and found that it significantly reduced the peripheral blood disease burden and prolonged the survival of leukemic mice (median survival of 60.5 vs 21 days, p = 0.0007). This screen for targetable pathways in T-ALL generated *in vitro* and *in vivo* preclinical data supporting NAMPT inhibition as a promising strategy for the treatment of T-ALL.

## Introduction

Recent advances in therapy have improved outcomes in T-cell acute lymphoblastic leukemia (ALL), which were historically inferior to B-ALL [1]. Outcomes in relapsed T-ALL however have remained poor, with 5-year overall survival <35% [1]. New strategies are thus needed to prevent and/or treat relapsed disease. Compounds targeting signaling pathways upregulated in T-ALL subtypes, including NOTCH, mTOR/PI3K, and JAK/STAT, have shown promise, and some are in current clinical trials [2–4]. In addition, inhibition of the glycolysis pathway through targeting NAMPT has shown efficacy in hematologic malignancies [5].

NAMPT aids in converting nicotinamide to nicotinamide mononucleotide, a rate-limiting step in the alternative salvage pathway for production of NAD^+^, a cofactor required in multiple pathways including glycolysis [6–8]. Since ATP generation through glycolysis is less efficient and therefore requires a higher rate of NAD/NADH redox reactions [9], cancer cells with rapid turnover and high bioenergetics requirements relying on NAD^+^ may be more sensitive to NAMPT inhibition [5], suggesting its therapeutic potential for cancer treatment. Knockout of NAMPT has been shown to reduce viability of acute myeloid leukemia [10] and colorectal cancer cells [11]. FK866, a non-competitive inhibitor of NAMPT, has been shown to reduce NAD+ levels and suppress glycolysis in human leukemia cells [12–14], reduce viability of both solid and hematologic cancers [5,9,15], and reduce leukemic disease burden *in vivo* [16].

Here, we identify targetable signaling pathways in T-ALL, and demonstrate the *in vitro* cytotoxicity of novel agents, including mTOR/PI3K inhibitors (gedatolisib, AZD2014, and LY3023414), G2M checkpoint inhibitors (AZD7762, PHA-793887, and AT7519) and NAMPT inhibitors (FK866 and STF-118804). We perform further testing of FK866 in PDX-expanded T-ALL cases of different molecular subtypes, providing preclinical evidence of both *in vitro* and *in vivo* cytotoxicity in T-ALL.

## Materials and methods

### Mice

*Kras*^*LSL-G12D/+*^.*Mb1*^*Cre/+*^ mice were generated as described by Junco et al [17]. NOD.Cg-Prkdc^scid^ Il2rg^tm1Wjl^/SzJ (NSG) mice were provided by Michele Redell (Baylor College of Medicine, BCM). Moribund mice were humanely sacrificed by isoflurane inhalation followed by cervical dislocation and bilateral thoracotomy. All animal experiments were performed with approval of the BCM Institutional Animal Care and Use Committee.

### Cell lines

The CEM (RRID:CVCL_0207) and HSB2 (RRID:CVCL_1859) human T-ALL lines were provided by H. Daniel Lacorazza (BCM) and Terzah Horton (BCM). The Jurkat (RRID:CVCL_0065) T-ALL line was obtained from ATCC (Manassas, VA, USA). All media components were obtained from Gibco (Waltham, MA, USA). Human cell lines were cultured in RPMI + L-glutamine supplemented with 20% fetal bovine serum (FBS) and 1% penicillin-streptomycin.

Mouse T-ALL cell lines (402, 428, 442) were derived from *Kras*^*LSL-G12D/+*^.*Mb1*^*Cre/+*^ leukemic mice. Thymus samples were homogenized and incubated in IMDM with GlutaMAX, containing 25 mM HEPES, 3.024 g/L sodium bicarbonate, 20% fetal bovine serum, 1% penicillin-streptomycin, 50 µM beta mercaptoethanol, 40 ng/mL IL-7, and 250 ng/mL Amphotericin B. Cultures which became established as immortalized cell lines retained a T-cell immunophenotype, and some became independent of IL-7 supplementation.

### RNA sequencing and GSEA

Thymus tissue from leukemic *Kras*^*LSL-G12D/+*^.*Mb1*^*Cre/+*^ mice and healthy age-matched control mice was collected and snap frozen. Samples were homogenized on liquid nitrogen using a mortar and pestle. Normal human thymus samples were obtained from Audubon Biosciences (Houston, TX, USA) and homogenized with a mortar and pestle as above. Human PDX T-ALL samples were obtained from banked cryopreserved samples previously isolated from the spleens of PDX mice. RNA was extracted from all samples using the AllPrep DNA/RNA mini kit from Qiagen (Hilden, DE).

RNA sequencing (RNA-Seq) of mouse samples was performed by Novogene (Beijing, CN) using STAR (version 2.5) [18] and HTSeq (version 0.6.1) [19] with the mm10 mouse genome (Novogene report date 2021-02-01). Counts were normalized with DeSeq2 [20]. Gene set enrichment calculations were completed using GSEA (v. 4.3.2) [21] for genes with adjusted p-values less than 0.05. GSEA was run with normalized counts and the default weighted scoring scheme. Statistics were estimated by 10K gene set permutations. The Broad-UC San Diego Molecular Signatures Database (MSigDB v2023.1.Hs) Hallmark [22] pathway sets were used, along with the v2031.1 mapping to human orthologs.

For evaluation of T-ALL PDX, RNA-Seq reads were aligned and quantified to human reference GRCh38 (GENCODE v32/Ensembl 98)(refdata-gex-GRCh38-2020-A dated 2020-07-07 downloaded from 10X Genomics, GENCODE gtf dated 2019-09-05) using RSEM (version 1.30) [23] with Bowtie2 (version 2.10) [24]. Expected counts were normalized with DeSeq2 [20]. Gene set enrichment calculations were completed using GSEA (v. 4.3.2) [21] for genes with adjusted p-values less than 0.05. GSEA was run in preranked mode with log2 fold change of normalized counts and the classic scoring scheme. Statistics were estimated by 10K gene set permutations. The Broad-UC San Diego Molecular Signatures Database (MSigDB v2023.1.Hs) Hallmark [22] pathway sets were used, along with the v2031.1 Ensembl gene mapping.

For evaluation of publicly-available T-cell lymphoblastic lymphoma (T-LBL) and thymus RNA-Seq data from GSE109231, raw counts were normalized with DeSeq2 [20].

### T-ALL PDX sample subtyping

Gene counts from the TARGET T-ALL dataset [3] were downloaded from the United States National Cancer Institute Genomic Data Commons Data Portal (Release date: 2022-03-29, Release Number: 32.0, Star alignment to GRCh38.d1.vd1, GENCODE v32 annotation). Batch effects between our T-ALL PDX samples and the 255 T-ALL samples in the TARGET data were addressed in R(4.2.2.) [25] using ComBat-seq [26] implemented within the SVA package [27] and using sex as a covariate. Twenty-one marker genes were selected based upon Dai et. al. [28] and Brady et. al. [29], including: *TLX1, TLX3, TAL1, TAL2, NKX2−1, LMO1, LMO2, MEF2C, SPI1,* and *HOX* family markers (*HOXA10, HOXA10-AS, HOXA11, HOXA13, HOXA2, HOXA3, HOXA5, HOXA6, HOXA7, HOXA9, HOXA-AS2, HOXA-AS3*).

### Chemicals and reagents

For *in vitro* studies, PHA-793887, AZD7762, FK866, STF-118804, gedatolisib, and doxorubicin were purchased from Selleckchem (Houston, TX, USA), and LY3023414, AZD2014, and AT7519 were purchased from MedChem Express (Monmouth Junction, NJ, USA). Drugs were dissolved in DMSO to 10 mM stocks, and stored at −80°C. For *in vivo* studies, FK866 (Selleckchem) was prepared to 20 mg/mL in DMSO and stored at −80°C, and freshly prepared at 1 mg/mL in 0.9% normal saline with solubilization by vortexing each day of treatment. NAD + was purchased from Cayman Chemical (Ann Arbor, MI, USA). Normal saline (0.9%) was obtained from Fisher (Waltham, MA, USA).

### Cell viability assays

For ATP assays for cell viability, human and mouse T-ALL cells were added to 96-well plates at 8x10^3^ or 2.5x10^3^ cells per well, and incubated with drug or 0.01% DMSO vehicle for 72 hours. PDX-expanded T-ALL cells were incubated with AZD7762 or FK866 for 48 hours. To evaluate for on-target activity of FK866, human T-ALL cell line CEM and mouse T-ALL cell line 402 were pre-treated or not with 200 µM NAD+ for 1 hour before treatment with indicated doses of FK866, doxorubicin, or 0.01% DMSO vehicle. ATP assays were conducted using CellTiter-Glo Luminescent Cell Viability Assay from Promega (Madison, WI, USA).

For flow cytometric assessment of cell viability, human T-ALL cell lines were added to 24-well plates at 1.5x10^4^ cells per well, and incubated with drug or 0.01% DMSO vehicle for 72 hours. Cells were stained with Annexin V-APC (BD Biosciences Cat# 550475, RRID:AB_2868885) (Becton Dickinson, Franklin Lakes, NJ, USA) and 7-AAD (eBioscience Cat# 00-6993-50) (San Diego, CA). Cells were analyzed on a LSRII flow cytometer (BD), and resulting data were interpreted with FlowJo software version 10.8.1 (BD).

### *In vivo* study

Locally banked diagnostic patient T-ALL samples from leukapheresis or bone marrow aspirates were utilized for xenografting. Banked cryopreserved samples of T-ALL PDX 105130 were thawed and injected via tail vein into male NSG mice at 5x10^6^ cells per mouse. Mice were monitored for leukemia engraftment weekly starting at 2 weeks after injection by evaluation of peripheral blood (PB) by flow cytometry using mouse anti-human CD5-FITC (BD Biosciences Cat# 555352, RRID:AB_395756) and CD45-PE (BD Biosciences Cat# 555483, RRID:AB_395875) (Becton Dickinson), with an LSRII flow cytometer and FlowJo software version 10.8.1 (BD). Once mice demonstrated >5% PB blasts, they were treated with either 20 mg/kg FK866 or vehicle (5% DMSO in 0.9% normal saline), 5 times weekly for 4 weeks via intraperitoneal injection. There was no significant difference in starting blast percentage between groups. PB blast percentage was assessed by flow cytometry weekly until mice became moribund and were humanely sacrificed. PB, spleen, and bone marrow cells from moribund leukemic mice were similarly assessed for for leukemic burden by CD5 and CD45 staining.

### Ethics statement

T-ALL patient samples were collected with informed written consent and children’s assent under protocols approved by the BCM Institutional Review Board, using archived samples and associated data that were accessed on August 1, 2021. IRB approval included approval for the authors’ access to patient identifiers associated with the samples.

### Statistics

IC_50_ values were determined after log-transforming and curve-fitting the data. Quantitative data for Annexin V/7-AAD viability studies and for expression of individual genes were analyzed by Student’s t-test. Differences in survival were analyzed with log-rank test. Statistical analyses were performed using GraphPad Prism version 9.4.1 (La Jolla, CA, USA).

## Results

### Similar targetable signaling pathways are upregulated in human and mouse T-ALL

**Fig 1:**
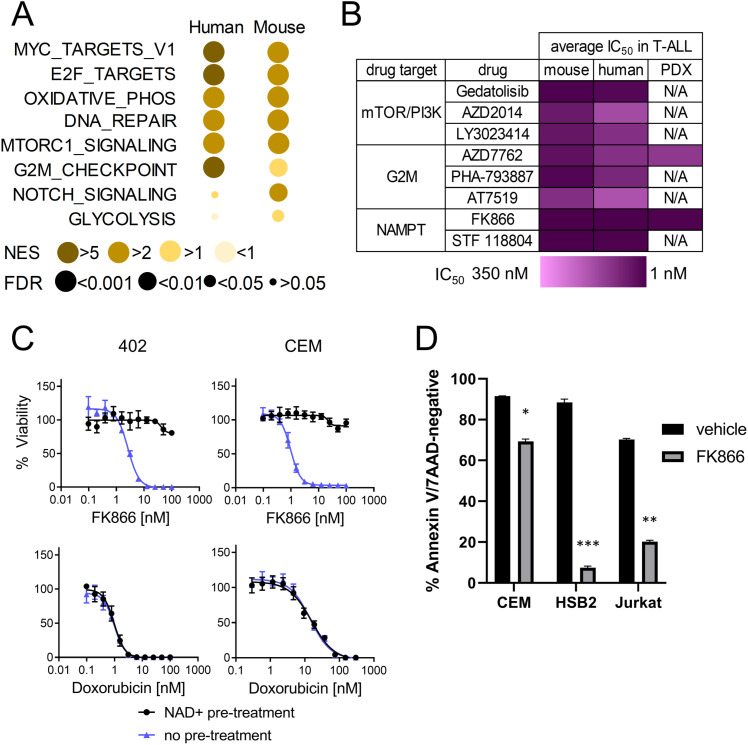
Drugs targeting upregulated signaling pathways effectively reduce the viability of mouse and human T-ALL cell lines. (A) Hallmark gene set enrichment analysis indicates similar gene sets are upregulated in T-ALL from both *Kras*^*LSL-G12D/+*^.*Mb1*^*Cre/+*^ mice (n = 5) and human T-ALL PDX samples (n = 4) compared to thymus control (n = 5 mouse, n = 2 human). NES indicated by circle color, FDR indicated by circle size. (B) Compounds targeting mTOR, G2M checkpoint, and glycolysis demonstrate low-nanomolar cytotoxicity in the mouse *Kras*^*LSL-G12D/+*^.*Mb1*^*Cre/+*^ T-ALL and human T-ALL cell lines, and AZD7762 and FK866 demonstrate low-nanomolar cytotoxicity in PDX cells. Dark boxes indicate lowest IC_50_ values, and each box indicates the average IC_50_ for three cell lines or PDX samples for each drug. (C) The effect of the most active inhibitor (FK866), but not a control cytotoxic agent (doxorubicin), is selectively reversed by NAD+ supplementation (black lines) in the mouse cell line 402 (left) and the human T-ALL line CEM (right), indicating FK866 functions by inhibiting NAMPT. (D) Low-nanomolar doses of FK866 induce apoptosis in human T-ALL lines, with a significant reduction of healthy, Annexin V-negative and 7-AAD-negative cells in CEM and HSB2 treated with 2 nM FK866, and in Jurkat treated with 5 nM FK866. Percentages indicate the average of two technical replicates per condition (*p < 0.05, **p < 0.01, ***p < 0.001).

We performed unsupervised hierarchical clustering of four diagnostic pediatric T-ALL samples with TARGET controls [3] and mapped them to distinct transcriptomic subtypes (S1 Table). We also performed RNA sequencing and GSEA to determine if our novel T-ALL mouse model, and our cohort of human T-ALL samples, feature key targetable signaling pathways previously implicated in human T-ALL [3,4,30]. Human PDX-expanded T-ALL cases (n = 4) were compared to healthy human thymus control samples (n = 2), and leukemic blasts from moribund *Kras*^*LSL-G12D/+*^.*Mb1*^*Cre/+*^ mice (n = 5) were compared to thymus cells from healthy control mice (n = 5). Five Hallmark gene sets were significantly upregulated (FDR < 0.05) in both our human and murine T-ALL cohorts, including E2F, G2M checkpoint, Myc, and mTOR signaling pathways, which have been shown to be involved in T-ALL pathogenesis [3,4,30]. Genes associated with the mitotic spindle were also significantly upregulated in the human T-ALL samples, and genes associated with glycolysis were significantly upregulated in the *Kras*^*LSL-G12D/+*^.*Mb1*^*Cre/+*^ leukemic blasts (Fig 1A). Additional pathways upregulated in the mouse and human samples compared to normal thymus are presented in S2 Table and S3 Table.

### Inhibitors of mTOR/PI3K, G2M checkpoint, and NAMPT/glycolysis are effective in human and murine T-ALL

We tested inhibitors of upregulated signaling pathways in human T-ALL cell lines and novel T-ALL cell lines derived from *Kras*^*LSL-G12D/+*^.*Mb1*^*Cre/+*^ mice. Inhibitors of mTOR/PI3K (gedatolisib, AZD2014, LY3023414), G2M checkpoint (AZD7762, PHA-793887, AT7519), and NAMPT/glycolysis (FK866, STF-118804) effectively reduced the viability of each T-ALL cell line at nanomolar-range doses. We also tested AZD7762 and FK866 in three of the PDX-expanded human T-ALL cases, including two from the *TAL1*/*LMO1* subtype and one from the *LYL1*/*LMO2* subtype, and both agents were effective in the nanomolar range (Fig 1B, S2 Fig, S3 Fig, and S4 Table). Overall, FK866 was the most cytotoxic agent tested, demonstrating an IC_50_ of less than 20 nM in each PDX-expanded T-ALL sample and T-ALL cell line. To confirm that FK866 cytotoxicity was mediated via NAMPT inhibition, we pre-treated the mouse T-ALL cell line 402 and the human T-ALL cell line CEM with NAD + , produced downstream of NAMPT. T-ALL lines pre-treated with NAD+ were resistant to FK866 but sensitive to doxorubicin, confirming that the observed rescue from cytotoxicity by NAD + pre-treatment was specific to the NAMPT inhibitor FK866 (Fig 1C). Finally, we confirmed the cytotoxic effect of FK866 in human T-ALL cell lines is mediated by apoptosis (Fig 1D and S4 Fig).

**Fig 2:**
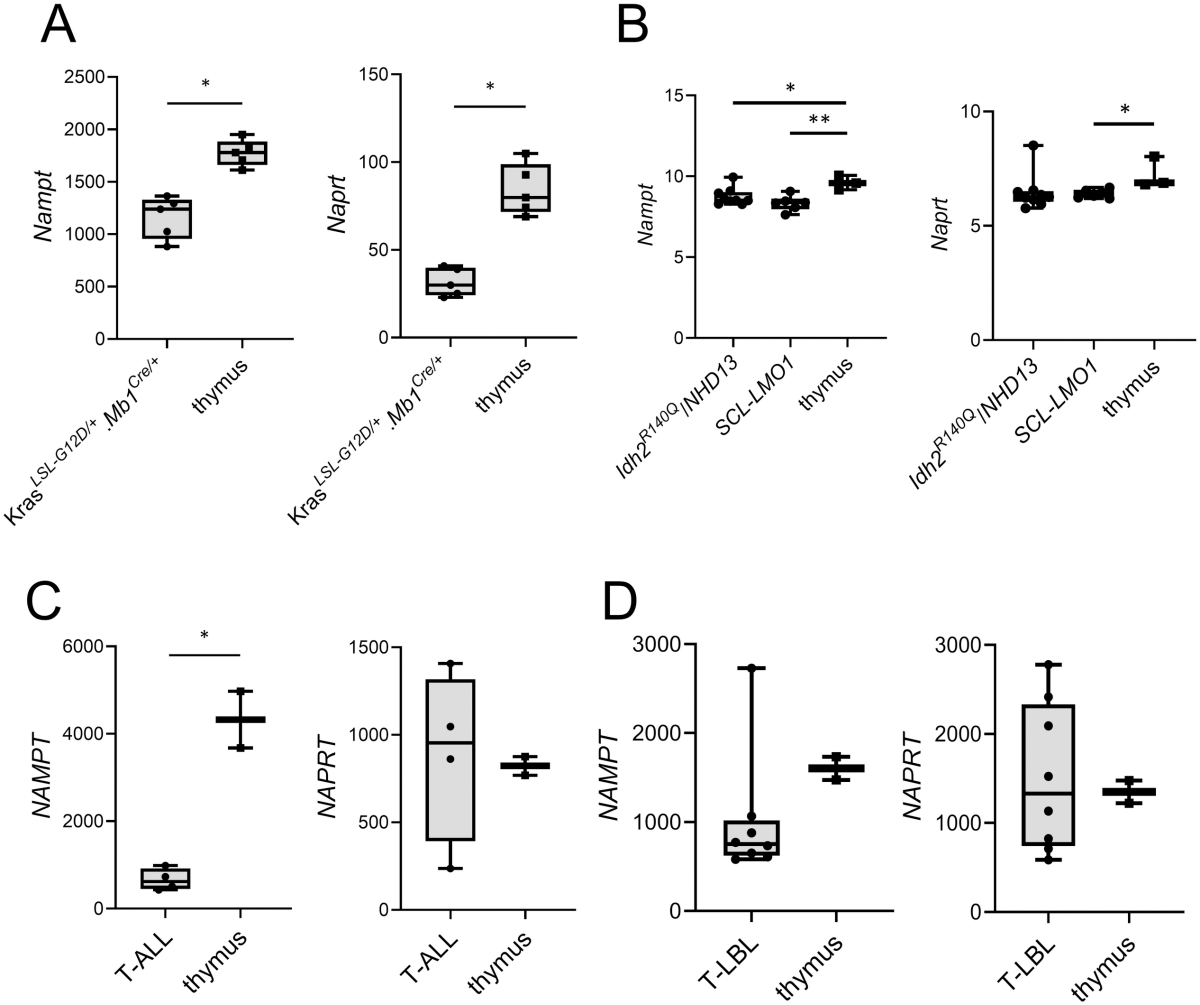
Mouse and human T-ALL samples show differential expression of genes involved in sensitivity to NAMPT inhibition. (A) Expression of enzymes involved in NAD+ biosynthesis, including *Nampt* and *Naprt*, is downregulated in mouse *Kras*^*LSL-G12D/+*^.*Mb1*^*Cre/+*^ T-ALL samples (n = 5) compared to thymus control (n = 5). Normalized counts are displayed (*p < 0.001). (B) *Nampt* is downregulated in other transgenic murine T-ALL models, *Idh2*^*R140Q*^/*NHD13* (n = 9) and *SCL*-*LMO1* (n = 6) mice, compared to thymus control (n = 3). Microarray expression values using normalized probe sets from publicly-available dataset GSE181007 are shown.. For *Nampt*, data for probe 1455320_at is shown. For *Naprt*, data for the only probe, 1454748_at, is shown (**p < 0.01, *p < 0.05). (C) *NAMPT* is downregulated in human T-ALL samples (n = 4) compared to thymus control (n = 2). Normalized counts are displayed (*p < 0.01). (D) There is a trend towards decreased *NAMPT* expression in pediatric T-LBL samples (n = 8) compared to thymus control (n = 2). Normalized RNA-Seq counts from publicly-available dataset GSE109231 are shown. Bars on box plots show the minimum and maximum individual values for each.

We also analyzed the mouse and human T-ALL and thymus control RNA-Seq data for the expression of *NAMPT/Nampt* and nicotinic acid phosphoribosyltransferase (*NAPRT/Naprt*), a rate-limiting enzyme similar to *NAMPT* which converts nicotinic acid to nicotinic acid mononucleotide, the first step in the alternative Preiss-Handler NAD synthesis pathway [6,8] (S1 Fig). Low expression of these genes has been previously reported to be associated with increased sensitivity to NAMPT inhibition [15,31,32]. Both human and mouse T-ALL samples displayed significantly downregulated *NAMPT*/*Nampt* expression, and mouse T-ALL had reduced expression of *Naprt*, compared to thymus control samples (Figs 2A and 2C). We also analyzed publicly-available gene expression data from two transgenic mouse models of T-ALL driven by different oncogenes which arrest at different T cell development stages, the *Idh2*^*R140Q*^/*NUP98-HOXD13* (*Idh2*^*R140Q*^*/NHD13*) and *SCL-LMO1* models [33], and gene expression data from pediatric T-LBL patient samples [34]. Similar to *Kras*^*LSL-G12D/+*^.*Mb1*^*Cre/+*^ mice, T-ALL samples from these mouse models have significantly decreased *Nampt* expression, and the *SCL*-*LMO1* model has decreased *Naprt* expression, compared to thymus control (Fig 2B and S5 Fig). The pediatric T-LBL cases displayed a trend towards decreased *NAMPT* expression compared to thymus control, and large intra-group variability in *NAPRT* expression, similar to our pediatric T-ALL cohort (Fig 2D).

### *In vivo* FK866 treatment prolongs survival of mice engrafted with a T-ALL PDX

**Fig 3:**
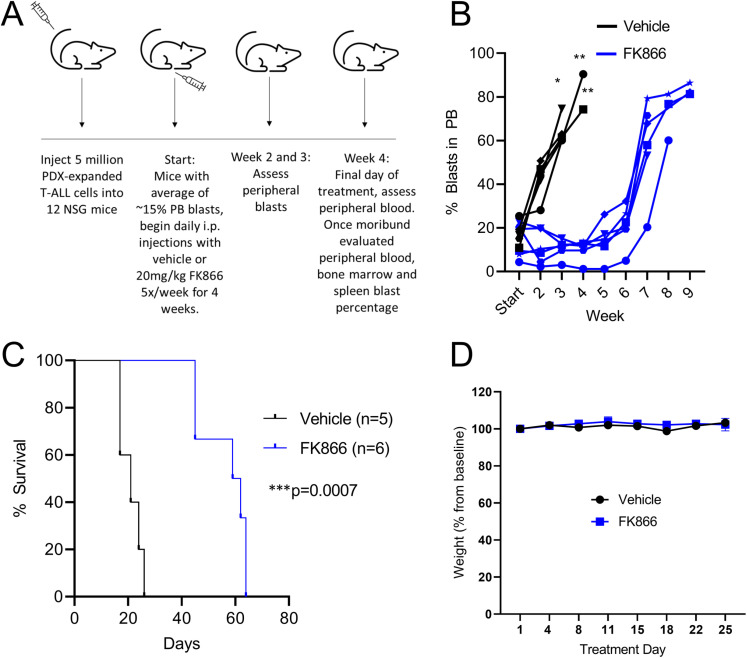
FK866 prolongs survival of mice xenografted with human T-ALL. (A) Experimental design schema. Mice were treated with FK866 20 mg/kg or vehicle by i.p. injection 5 days per week for 4 weeks. (B) Treatment with FK866 (blue) delayed the rise in PB blast percentage compared to vehicle (black) (*p < 0.001, **p < 0.0001 at indicated timepoint, Student’s t-test). (C) Mice treated with FK866 demonstrated significantly prolonged survival (median of 60.5 vs 21 days from treatment start, ***p = 0.0007). (D) Mice treated with FK866 demonstrated no weight loss or other apparent signs of toxicity. Mean mouse weights with standard deviation are shown.

Because FK866 was the most effective compound in our *in vitro* testing, we chose it for *in vivo* testing in T-ALL xenografted mice. We treated NSG mice engrafted with PDX-expanded sample 105130 with either vehicle or FK866 (experimental schema in Fig 3A). When each mouse showed >5% PB blasts we randomized them to receive either vehicle or FK866, with no significant difference in starting leukemia percentage (18% in vehicle vs 14% in FK866, p = 0.43). We treated mice daily 5 days per week for 4 weeks, with the exception of three vehicle mice that died of disease progression during the final week of treatment, and measured PB blast percentage at weekly intervals starting with the second week of treatment. FK866 treated mice showed an average decrease of 4% in PB burden over the 4-week treatment course compared to an average rise of 55% in vehicle treated mice (p < 0.0001). The PB blast percentage in the FK866-treated mice did not substantially increase until 3 weeks after the end of treatment (Fig 3B).

Mice treated with FK866 demonstrated a significant survival benefit compared to vehicle-treated mice, with median survival from treatment initiation of 60.5 days (range 45–64) versus 21 days (range 17–26 days; p = 0.0007, Fig 3C). Necropsy studies of PB, spleen, and bone marrow demonstrated high leukemic burdens in all mice at time of death (S6 Fig). No significant toxicities were observed in the mice treated with FK866, and weights in both cohorts remained stable throughout treatment (Fig 3D).

## Discussion

We identified upregulation of the E2F, G2M checkpoint, Myc, and mTOR signaling pathways in both human T-ALL and our *Kras*^*LSL-G12D/+*^.*Mb1*^*Cre/+*^ murine T-ALL model. Glycolysis was also significantly upregulated in blasts from our murine model. These pathways have previously been implicated in T-ALL etiology and/or response to Notch-targeting therapies [3,4,30,35], indicating the pertinence of our *Kras*^*LSL-G12D/+*^.*Mb1*^*Cre/+*^ murine model and T-ALL PDX sample cohort for testing therapies which may have improved efficacy against T-ALL. We further demonstrated that inhibition of mTOR/PI3K, G2M checkpoint, or the glycolysis pathway through NAMPT inhibition had potent *in vitro* anti-leukemic activity in human and murine T-ALL cell lines, and inhibitors of G2M checkpoint and NAMPT were effective in human PDX-expanded T-ALL samples of different molecular subtypes. Furthermore, the NAMPT inhibitor FK866 significantly prolonged the survival of mice xenografted with a human T-ALL sample *in vivo*, without observed toxicities.

Of note, multiple studies that profiled cancer cells to identify those with sensitivity to NAMPT inhibition found that cells with lower NAMPT expression demonstrated increased sensitivity [31,32]. Our expression profiling of both human and mouse T-ALL samples, and publicly available human T-LBL [34] and mouse T-ALL expression data [33], suggest *NAMPT* expression is downregulated in T cell malignancies, likely explaining the efficacy of FK866 that we observed in these samples. Additionally, cancer cell lines with low expression of *NAPRT* allowed for high dose treatment of FK866 and *in vivo* rescue of xenografted mice with nicotinic acid, the precursor to NAD biosynthesis via *NAPRT* (see S1 Fig), without sacrificing the therapeutic potential [9]. Another first-generation NAMPT inhibitor, GMX1777, also displayed an improved therapeutic index against cancer cells with low *NAPRT* expression *in vivo*, when administered at high doses with nicotinic acid rescue [32]. *Naprt* expression was significantly downregulated in blasts from our mouse T-ALL model, and *NAPRT* expression is decreased in a subset of pediatric T-ALL and T-LBL cases, suggesting these patients may benefit from first-generation NAMPT inhibitors like FK866 or GMX1777 in combination with nicotinic acid rescue. This stands in contrast to a second-generation NAMPT inhibitor, OT-82, which is also effective against multiple leukemias, but more cytotoxic in cells with higher *NAPRT* expression [36], and requires a reduction of dietary nicotinic acid to improve the therapeutic index against leukemia *in vivo* [37], a strategy which may be limited by patient adherence. This suggests that unlike FK866 or GMX1777, the therapeutic index of OT-82 in cases with low *NAPRT* expression will not be improved by simple nicotinic acid co-administration. The potential use of nicotinic acid to prevent toxic side effects, while maintaining anti-leukemic efficacy of first-generation NAMPT inhibitors like FK866, offers a treatment strategy not yet trialed in human studies.

FK866 was first tested clinically in a phase I trial in 24 patients with advanced solid tumors [38]. The most common toxicity was dose limiting thrombocytopenia as well as mild fatigue and nausea. Since then, there have been three other Phase I/II clinical trials evaluating the use of FK866 for the treatment of refractory B-cell chronic lymphocytic leukemia (NCT00435084), advanced melanoma (NCT00432107), and cutaneous T-cell lymphoma (CTCL) (NCT00431912) [39]. In general, studies of FK866 have demonstrated suboptimal efficacy and mild to moderate adverse effects, primarily gastrointestinal side events and cytopenias including mild lymphopenia. Additionally, human lymphocytes are resistant to doses of FK866 which effectively kill human T-ALL cell lines and T-ALL PDX used in this study [40], suggesting careful dosing of FK866 could spare normal human lymphocytes while maintaining anti-leukemic efficacy. Alternative NAMPT-targeting approaches, such as inhibitors with greater specificity; combination therapy; dual inhibitors; antibody-drug conjugates to deliver the drug specifically to cancer cells; and NAMPT-targeting proteolytic chimeras combined with nicotinic acid rescue, are in development [41,42]. Currently there is one active Phase I trial of the dual PAK4/NAMPT inhibitor KPT-9274, in patients with relapsed and refractory acute myeloid leukemia (NCT04914845).

Here, we identified common dysregulated pathways in the *Kras*^*LSL-G12D/+*^.*Mb1*^*Cre/+*^ T-ALL model, which demonstrated the applicability of this model to T-ALL signaling and inhibitor studies. We also illustrated the preclinical efficacy of inhibitors of several pathways, particularly NAMPT. Finally, our findings were consistent across a range of samples, including PDX-expanded primary samples and immortalized murine and human cell lines. Further study of NAMPT inhibition holds promise to improve outcomes in childhood T-ALL and other malignancies.

## Supporting information

S1 Fig NAMPT and NAPRT contribute to NAD synthesis, and NAMPT is inhibited by FK866. Diagram showing the synthesis of NAD, a cofactor necessary for glycolysis, via pathways involving NAMPT (salvage pathway) and NAPRT (Preiss-Handler pathway). FK866 is an inhibitor of NAMPT. Nicotinamide (NAM), nicotinamide phosphoribosyltransferase (NAMPT), nicotinamide mononucleotide (NMN), nicotinamide mononucleotide adenine transferase (NMNAT), nicotinic acid (NA), nicotinic acid phosphoribosyltransferase (NAPRT), nicotinate mononucleotide (NAMN), NAD synthase (NADSYN), nicotinamide adenine dinucleotide (NAD), glyceraldehyde 3-phosphate (G3P), glyceraldehyde 3-phosphate dehydrogenase (G3PDH), 1,3-bisphosphoglycerate (13BPG).
(TIF)

S2 Fig Mouse T-ALL cell lines are sensitive to drugs targeting upregulated signaling pathways. *Kras*^*LSL-G12D/+*^.*Mb1*^*Cre/+*^ T-ALL cell lines, identified by the legend on the lower right, were incubated with compounds targeting mTOR (gedatolisib, AZD2014, LY3023414), G2M checkpoint (AZD7762, PHA793887, AT7519), or glycolysis (FK866, STF118804), with three technical replicates per data point, for 72 hours before viability was measured by ATP assay. Each drug demonstrated nanomolar-range cytotoxicity, with glycolysis inhibitors demonstrating the most cytotoxic effect. IC_50_ values are provided in Table S4.
(TIF)

S3 Fig Human T-ALL cell lines and T-ALL PDX are sensitive to drugs targeting upregulated signaling pathways. (A) Human T-ALL cell lines, identified by the legend on the lower right, were incubated with compounds targeting mTOR (gedatolisib, AZD2014, LY3023414), G2M checkpoint (AZD7762, PHA793887, AT7519), or glycolysis (FK866, STF118804), with three technical replicates per data point, for 72 hours before viability was measured by ATP assay. Each drug demonstrated nanomolar-range cytotoxicity, with glycolysis inhibitors demonstrating the most cytotoxic effect. IC_50_ values are provided in Table S4. (B) Pediatric T-ALL PDX samples, identified by the legend on the right, were incubated with compounds targeting G2M checkpoint (AZD7762) or glycolysis (FK866) for 48 hours, with three technical replicates per data point, before viability was measured by ATP assay. Both drugs demonstrated low-nanomolar cytotoxicity in each sample. IC_50_ values are provided in Table S4.
(TIF)

S4 Fig FK866 induces apoptosis in human T-ALL cell lines. Low-nanomolar doses of FK866 induce apoptosis in human T-ALL lines, with a significant reduction of healthy, Annexin V-negative and 7-AAD-negative cells in CEM and HSB2 treated with 2 nM FK866, and in Jurkat treated with 5 nM FK866. Representative dot plots shown.
(TIF)

S5 Fig Mouse T-ALL samples show differential expression of genes involved in sensitivity to NAMPT inhibition. *Nampt* is downregulated in other transgenic murine T-ALL models, *Idh2*^*R140Q*^/*NHD13* (n = 9) and *SCL*-*LMO1* (n = 6) mice, compared to thymus control (n = 3). Microarray expression values using normalized probe sets from publicly-available dataset GSE181007 are shown, using *Nampt* probes 1417190_at (left) and 1448607_at (right). Bars on box plots show the minimum and maximum individual values for each.
(TIF)

S6 Fig There is no difference in disease burden of moribund leukemic mice previously treated with vehicle vs FK866. NSG were treated with FK866 20 mg/kg or vehicle by i.p. injection 5 days per week for 4 weeks. Tissues were collected from moribund leukemic mice, at a median of 60.5 days (FK866) vs 21 days (vehicle) from treatment start, and analyzed for CD19 + percentage by flow cytometry. There was no difference in disease burden for spleen, bone marrow (BM), or peripheral blood (PB) between the groups when mice were moribund with leukemia.
(TIF)

S1 Table Characteristics of diagnostic pediatric T-ALL PDX samples and normal thymus controls. (XLSX)

S2 Table Upregulated Hallmark signaling pathways in human T-ALL PDX samples (n = 4) compared to thymus controls (n = 2). Abbreviations: NES, normalized enrichment score; NOM, nominal; FDR, false discovery rate; FWER, family-wise error rate.
(XLSX)

S3 Table Upregulated Hallmark signaling pathways in mouse T-ALL samples (n = 5) compared to thymus controls (n = 5). Abbreviations: NES, normalized enrichment score; NOM, nominal; FDR, false discovery rate; FWER, family-wise error rate.
(XLSX)

S4 Table IC_50_ values (in nM) for pathway inhibitors in mouse and human T-ALL cell lines and pediatric T-ALL PDX samples. (XLSX)
