# Supplementary figures and images for: Efficacy of NAMPT inhibition in T-cell acute lymphoblastic leukemia
Source: PLoS One. 2025 Jun 17;20(6):e0324443. doi: 10.1371/journal.pone.0324443 (PMC12173385; doi:10.1371/journal.pone.0324443)

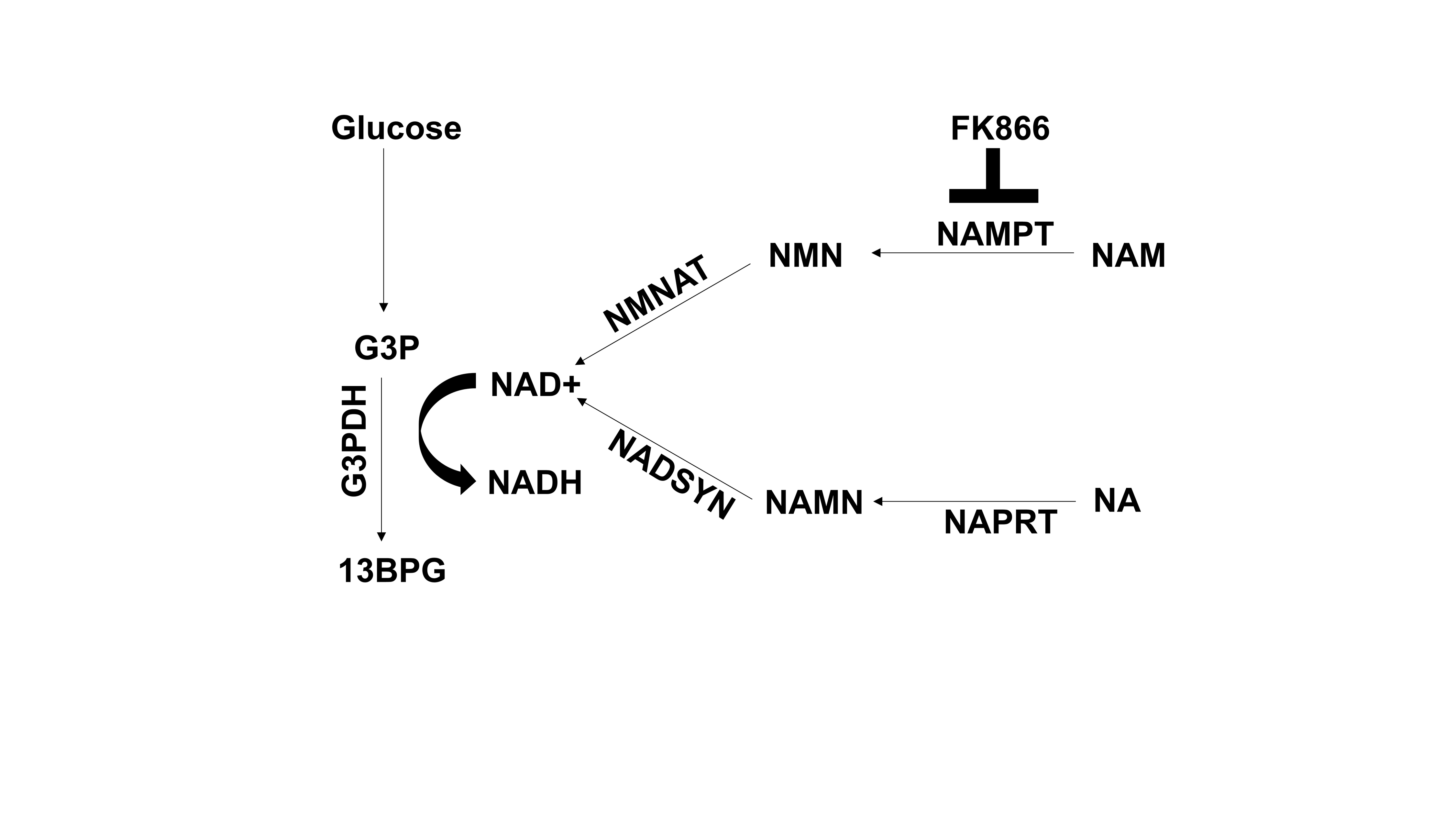

Supplement: S1 Fig — Diagram showing the synthesis of NAD, a cofactor necessary for glycolysis, via pathways involving NAMPT (salvage pathway) and NAPRT (Preiss-Handler pathway). FK866 is an inhibitor of NAMPT. Nicotinamide (NAM), nicotinamide phosphoribosyltransferase (NAMPT), nicotinamide mononucleotide (NMN), nicotinamide mononucleotide adenine transferase (NMNAT), nicotinic acid (NA), nicotinic acid phosphoribosyltransferase (NAPRT), nicotinate mononucleotide (NAMN), NAD synthase (NADSYN), nicotinamide adenine dinucleotide (NAD), glyceraldehyde 3-phosphate (G3P), glyceraldehyde 3-phosphate dehydrogenase (G3PDH), 1,3-bisphosphoglycerate (13BPG). (TIF) [file pone.0324443.s001.tif]

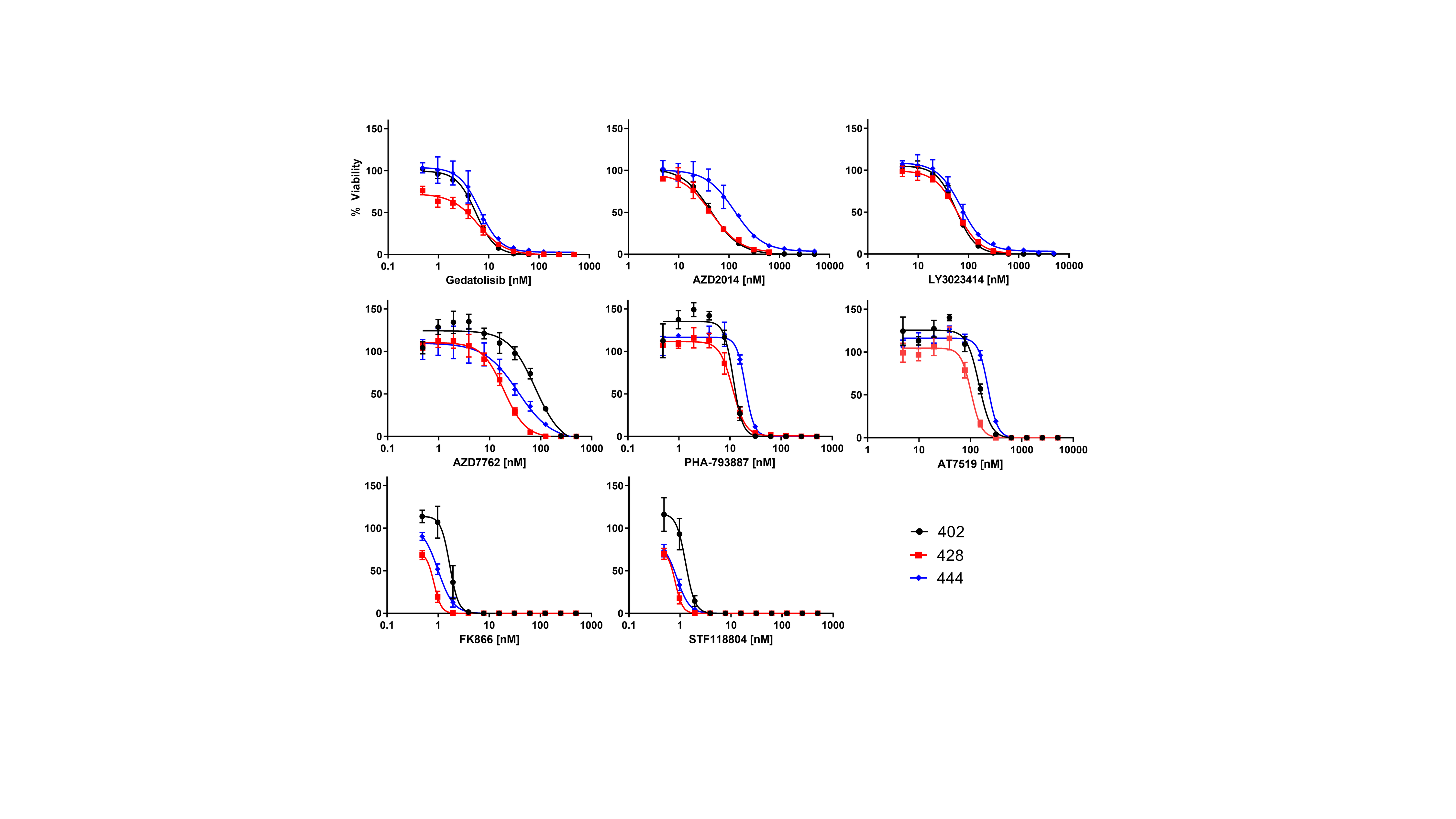

Supplement: S2 Fig — KrasLSL-G12D/+.Mb1Cre/+ T-ALL cell lines, identified by the legend on the lower right, were incubated with compounds targeting mTOR (gedatolisib, AZD2014, LY3023414), G2M checkpoint (AZD7762, PHA793887, AT7519), or glycolysis (FK866, STF118804), with three technical replicates per data point, for 72 hours before viability was measured by ATP assay. Each drug demonstrated nanomolar-range cytotoxicity, with glycolysis inhibitors demonstrating the most cytotoxic effect. IC50 values are provided in Table S4. (TIF) [file pone.0324443.s002.tif]

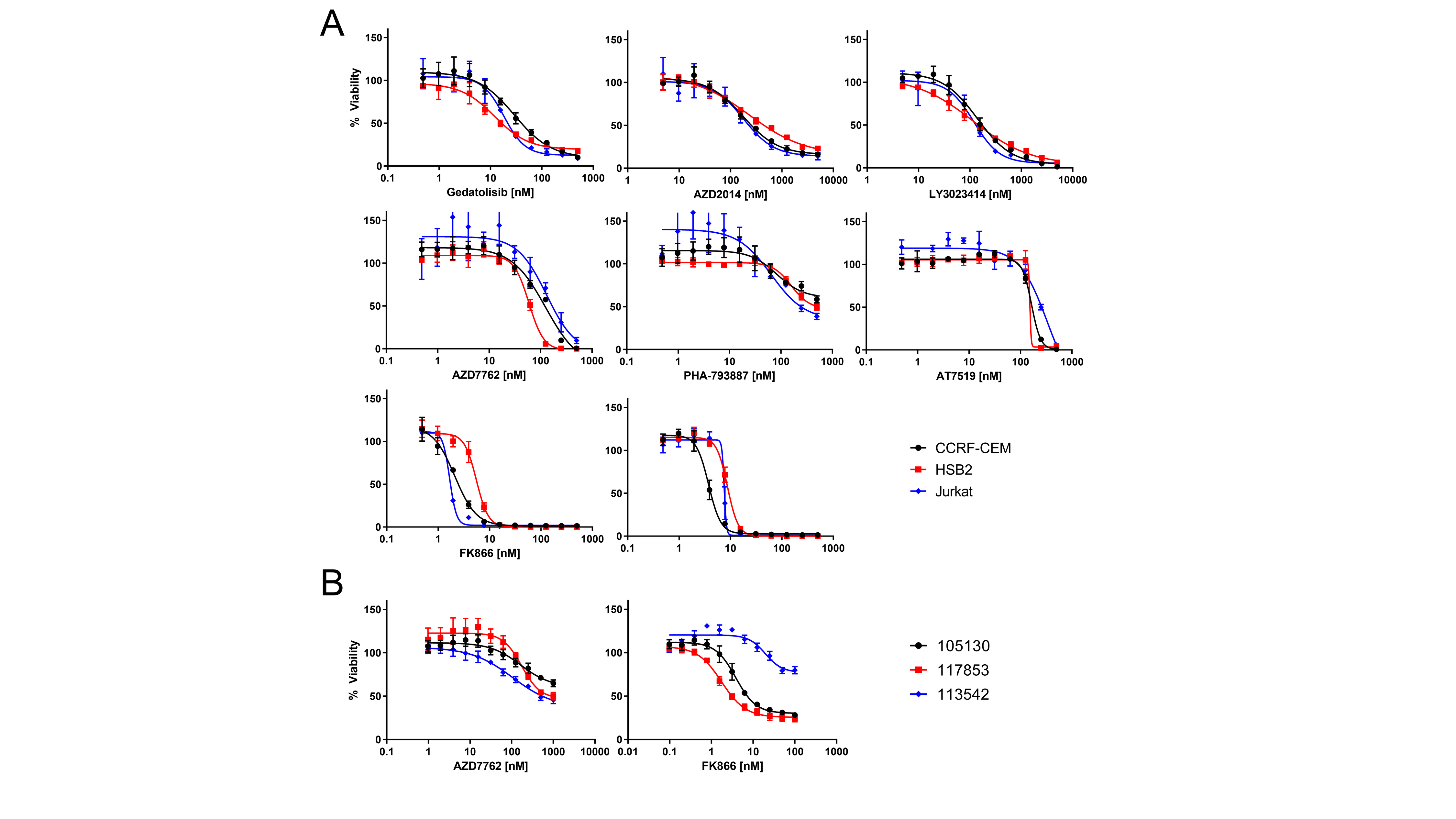

Supplement: S3 Fig — (A) Human T-ALL cell lines, identified by the legend on the lower right, were incubated with compounds targeting mTOR (gedatolisib, AZD2014, LY3023414), G2M checkpoint (AZD7762, PHA793887, AT7519), or glycolysis (FK866, STF118804), with three technical replicates per data point, for 72 hours before viability was measured by ATP assay. Each drug demonstrated nanomolar-range cytotoxicity, with glycolysis inhibitors demonstrating the most cytotoxic effect. IC50 values are provided in Table S4. (B) Pediatric T-ALL PDX samples, identified by the legend on the right, were incubated with compounds targeting G2M checkpoint (AZD7762) or glycolysis (FK866) for 48 hours, with three technical replicates per data point, before viability was measured by ATP assay. Both drugs demonstrated low-nanomolar cytotoxicity in each sample. IC50 values are provided in Table S4. (TIF) [file pone.0324443.s003.tif]

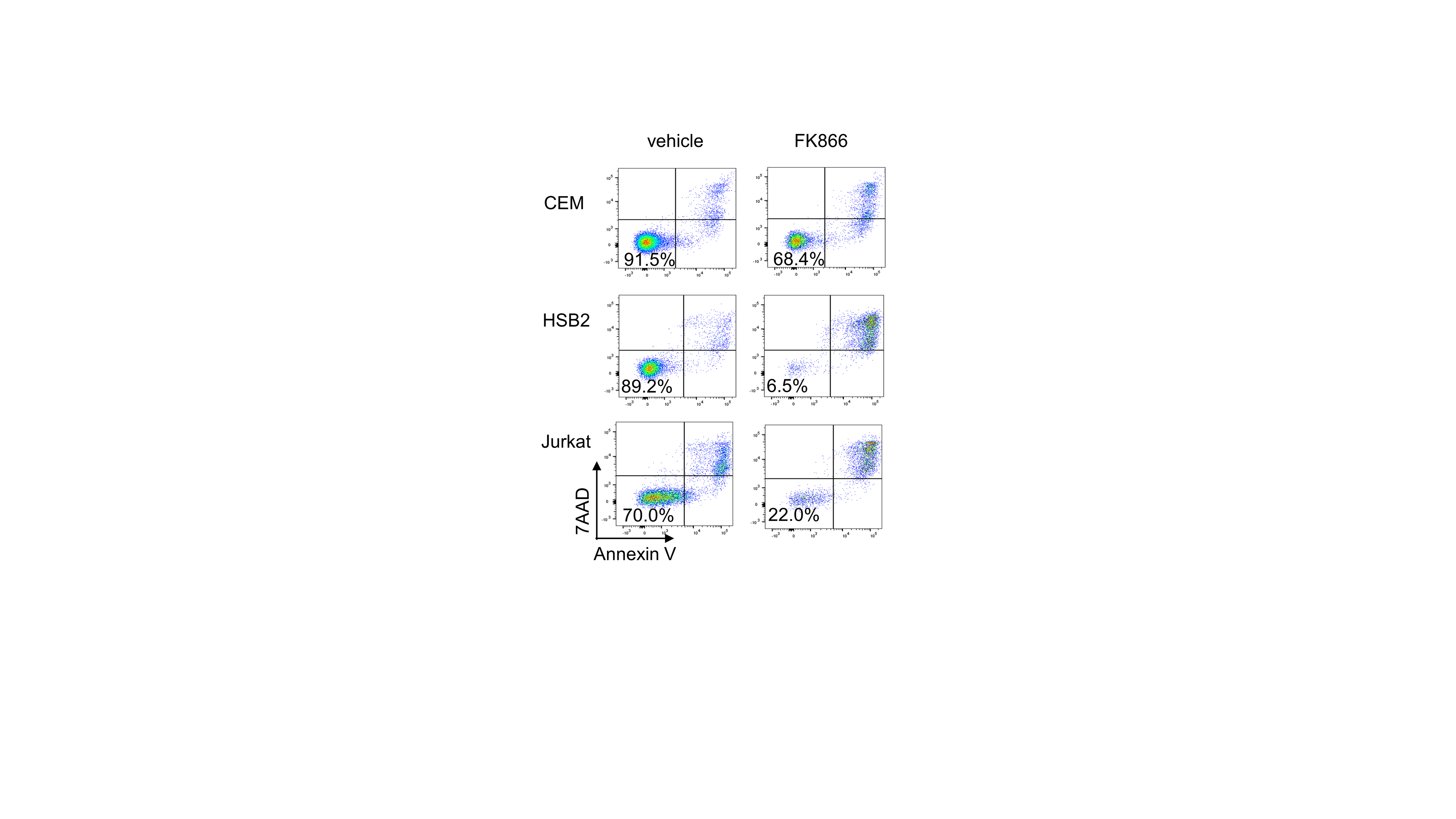

Supplement: S4 Fig — Low-nanomolar doses of FK866 induce apoptosis in human T-ALL lines, with a significant reduction of healthy, Annexin V-negative and 7-AAD-negative cells in CEM and HSB2 treated with 2 nM FK866, and in Jurkat treated with 5 nM FK866. Representative dot plots shown. (TIF) [file pone.0324443.s004.tif]

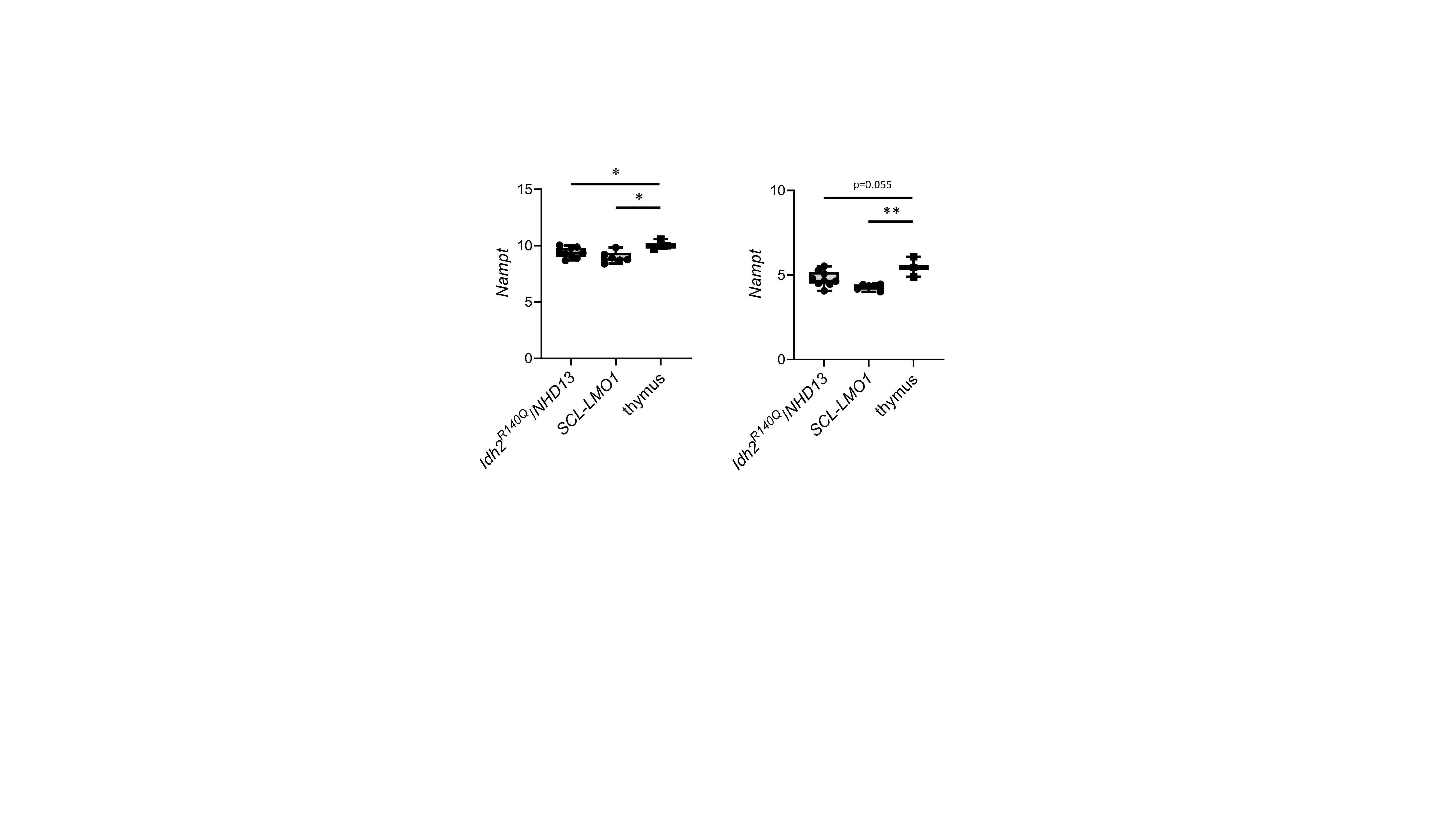

Supplement: S5 Fig — Nampt is downregulated in other transgenic murine T-ALL models, Idh2R140Q/NHD13 (n = 9) and SCL-LMO1 (n = 6) mice, compared to thymus control (n = 3). Microarray expression values using normalized probe sets from publicly-available dataset GSE181007 are shown, using Nampt probes 1417190_at (left) and 1448607_at (right). Bars on box plots show the minimum and maximum individual values for each. (TIF) [file pone.0324443.s005.tif]

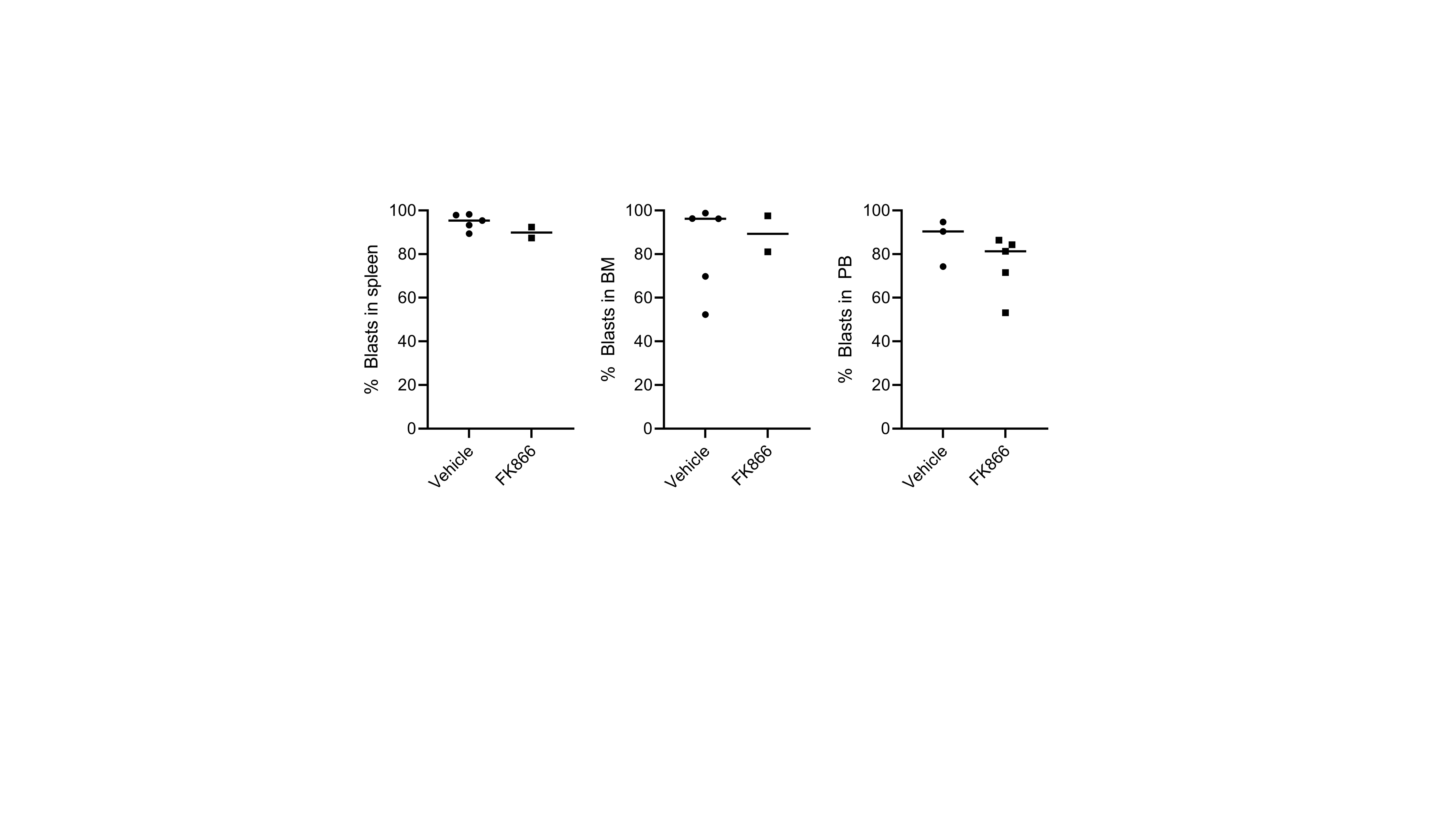

Supplement: S6 Fig — NSG were treated with FK866 20 mg/kg or vehicle by i.p. injection 5 days per week for 4 weeks. Tissues were collected from moribund leukemic mice, at a median of 60.5 days (FK866) vs 21 days (vehicle) from treatment start, and analyzed for CD19 + percentage by flow cytometry. There was no difference in disease burden for spleen, bone marrow (BM), or peripheral blood (PB) between the groups when mice were moribund with leukemia. (TIF) [file pone.0324443.s006.tif]
